# Equivalent circuit of a silicon–lithium p–i–n nuclear radiation detector

**DOI:** 10.1038/s41598-023-39710-5

**Published:** 2023-08-02

**Authors:** Ahmet Saymbetov, Ramizulla Muminov, Zhang Jing, Madiyar Nurgaliyev, Nursultan Japashov, Yorkin Toshmurodov, Nurzhigit Kuttybay, Ainur Kapparova, Batyrbek Zholamanov, Sayat Orynbassar, Nursultan Koshkarbay

**Affiliations:** 1 Al-Farabi Kazakh National University, Almaty, Kazakhstan; 2 Physical-Technical Institute, Uzbekistan Academy of Sciences, Tashkent, Uzbekistan; 3 Tashkent Institute of Irrigation and Agricultural Mechanization Engineers, Tashkent, Uzbekistan

**Keywords:** Electronics, photonics and device physics, Techniques and instrumentation, Physics

## Abstract

Nuclear radiation detectors are indispensable for research in the field of nuclear radiation, X-ray spectroscopy and other areas. Interest in silicon p–i–n detectors of nuclear radiation is increasing today due to the possibility of their operation under normal conditions. In this paper, an equivalent circuit of a silicon–lithium p–i–n nuclear radiation detector is proposed. The proposed circuit is obtained using the classical Shockley equation for silicon semiconductors and the telegraph equations. The parameters of the equivalent circuit were determined using the multiple regression method. As a result of simulation of the model in the MATLAB Simulink graphical development environment, the amplitude-frequency and phase-frequency characteristics of the proposed model were obtained. Using the Monte Carlo method, the alpha-decay of the uranium isotope ${}_{92}{}^{233}\mathrm{U}$, thorium isotope ${}_{90}{}^{227}\mathrm{Th}$ and americium isotope ${}_{95}{}^{241}\mathrm{Am}$ the alpha-decay spectrum was obtained. Obtained alpha-decay spectra coincides with the experimental data, presented in previous works of other authors.

## Introduction

Semiconductor p–i–n structured detectors are used in many fields of research as precision instruments^1^, especially in high-energy physics experiments^2^. The appearance of detectors with a larger detection area generated great interest in them because they significantly improved the efficiency of the detectors and made it possible to register weakly intense charged particles^3^. However, today, despite the fact that the physical processes in p–i–n diodes and their characteristics have been well studied, scientists are still working on developing large-sized semiconductor detectors based on p–i–n structures^4–6^. Large sized Si(Li) detectors are used in medical imaging, high-energy astrophysics, Compton polarimetry, monitoring of nuclear waste^7^ The main problems in improving p–i–n detectors of large sizes are related to its development technology^8,9^ and the development of optimal readout electronics for these detectors^10,11^. In^12,13^ authors showed application of silicon p–i–n diodes for spectroscopy. Equivalent circuit of a p–i–n diode was presented and preamplification noise was investigated.

Dementyev et al.^14^ in their work broadly studied the readout electronics of p–i–n detectors. In their work, authors brought valuable evidence about the pros and cons of p–i–n diodes as X–ray detectors. As an advantage of p–i–n detectors, they emphasize the following characteristics: resistance to the magnetic field; compact size; low operating voltage; inherent stability, and long lie time. As disadvantages of the p–i–n detectors, the authors mentioned the following characteristics: domain energy resolutions of p–i–n detectors are at low energies therefore they need a high gain preamplifier system relatively poor timing resolution and problems related to accepting high counting rate. A number of these problems were solved by some groups of authors, for instance, Muminov et al.^15,16^ proposed a unique technology for the fabrication of large-sized Si(Li) p–i–n detectors with help of double-sided diffusion and drift of Li ions into monocrystalline silicon. Applying this technology authors could obtain large-sized Si(Li) p–i–n detectors, where they could increase the counting rate of the detector due to its size and did increase its efficiency due to the uniform distribution of Li ions in the i- region. Mostly used technology for increasing the count rate and resolution of detectors is to use various cooling technologies^17,18^ during detector operation. In order to achieve a high-speed counting rate Gontard et al.^19^, have designed a high‐bandwidth circuit at the expense of electronic noise and used a prototype of the electronic circuit connected to a detector, aiming to detect single electron events of 200 keV.

Elshennawy and Sunil^20^ in their recent work described the architecture and device of p–i–n diode detectors for gamma radiation. In their work, they use a model of the simple diode in order to simulate signals from gamma rays. They divided the array of p–i–n diodes into clusters, where each cluster had ten p–i–n diodes. These clusters were used to create the sensitive area of the detector. As a result of their work, the authors show that their diode had a very good discriminating ability, for a 20 keV in particle energy the dynamic differential value for the diode was as high as 12 mV. Authors in^21,22^ demonstrated evaluation of dielectric properties of semiconductors. In^23^ authors considered changes of semiconductor’s dielectric properties after high gamma irradiation doses.

The above articles show a high interest in this topic and a large number of studies are devoted to Si(Li) p–i–n detectors of the gamma and X-ray range. Although the scientific interest in this area is quite large^9,24^, its amount is not sufficient to meet practical purposes.

One of the most important characteristics of any electrical devices is their accurate equivalent circuit. There are various equivalent models have been developed for p–i–n photodiodes, close relative to Si(Li) p–i–n detectors, that allow predicting the behavior of a semiconductor device under different conditions^25–27^. The equivalent circuit of a semiconductor detector was proposed in the works^28,29^. In these works, a charge separation method is proposed for determining the coordinates of a charged particle hit. In the work^30^ modeling p–i–n detector of nuclear radiation in SILVACON development environment. A simulation of the manufacture of a p–i–n structure was made and a simulation of the operation of the detector was carried out. The simulation is based on the classic Shockley equation for an ideal diode. In the work^31^ equivalent circuit of p–i–n structure under reverse bias was developed. In this work Poisson’s continuity equation to determine charge carrier concentration. The paper also presents an equivalent circuit of a p–i–n structure in the form of an RC chain of n links and a study of the dependence of resistance and capacitance on the magnitude of the reverse voltage. Authors of the work^32^ reviewed application of p–i–n diodes for gamma and X-ray detection. In this paper, the main attention is paid to the scheme of charge collection and preamplification of the detector signal.

In our recent papers^15,33^, we proposed a new method of obtaining large-sized Si(Li) p–i–n detectors and investigated the physical processes during the formation of the i-region. In order to deeply explain the processes of the newly obtained detector here, in the current work, we proposed modeling and designing a signal formation procedure in these detectors using the classical Shockley equation for silicon semiconductors and a system of telegraph equations. This paper shows the use of the multiple regression method to determine the values of the equivalent circuit elements using the telegraph equation. The resulting model was simulated and the spectra of alpha particles were obtained during the decay of some isotopes.

When designing automatic readout electronics for detectors, it is necessary to take into account the behavior of the system in various operating conditions. For this purpose, this paper shows the simulation of p–i–n detectors of nuclear radiation by the equivalent substitution method. The silicon–lithium nuclear radiation detector is a semiconductor with a p–i–n structure. Works^34–36^ show equivalent circuits of p–i–n diodes, which are the baseline for our research. In^33^ we showed the distribution of lithium ions in a silicon crystal under the action of a homogeneous electric field during the creation of the detector. Here our task is to simulate the response of the p–i–n structure to external excitation using an equivalent transformation. In this paper, the modeling process can be divided into three stages: modeling the reverse current through a semiconductor detector at the moment of radiation detection, determining the parameters of an equivalent circuit based on a telegraph equation, and simulating the operation of an equivalent circuit. The first stage consists of an approximate representation of the detector in the form of an ideal diode under reverse bias conditions. Then such a diode is described by the Shockley equation, and the generation of charge carriers is described by the continuity equation taking into account recombination and ionization. At the second stage, the found function will be equated to the classical telegraph partial differential equation for current. At the third stage, a simulation of the resulting equivalent circuit will be made.

## Modeling response of p–i–n structure on nuclear radiation

**Figure 1:**
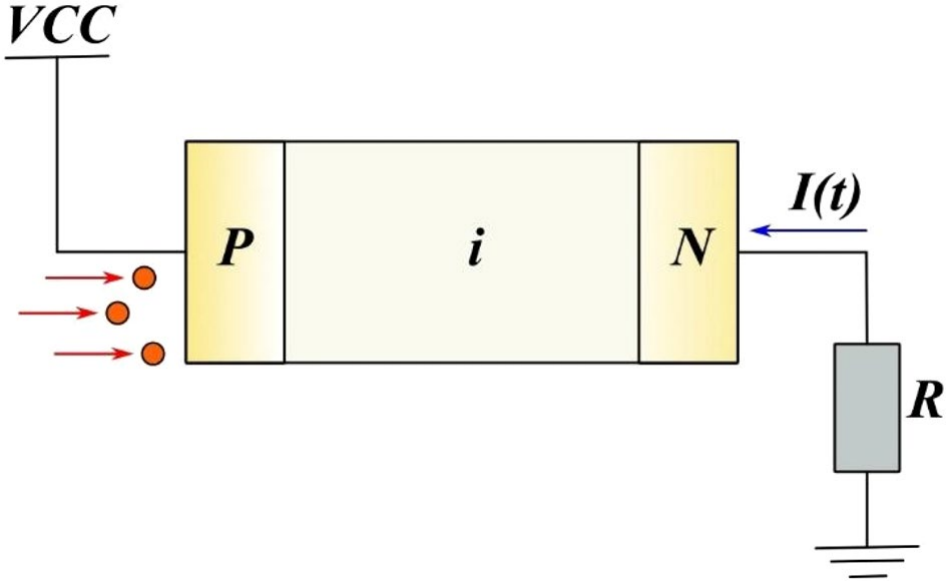
Electric circuit of p–i–n detector.

Semiconductor silicon Si(Li) detectors are used to obtain alpha, beta and gamma radiation spectra. Let's consider a model of a semiconductor diode with different conductivity of p, n and i-regions. When a semiconductor device is connected to reverse bias, a reverse current occurs in it, as shown in Fig. 1.

1 $$U\left(t\right)=V-RI(t)$$

Figure 1 shows the electrical circuit of a semiconductor nuclear radiation detector in operating mode. The voltage V is shifted in the opposite direction to expand the sensitive area. The voltage incident on the detector at the reverse bias can be calculated from the expression (1). The reverse current is obtained from Ohm’s law in differential form (2). The current is created by minority charge carriers in the p, n and i-regions

2 $$j\left(x,t\right)={q}_{e}{n}_{p}\left(x\right){v}_{n}+ {q}_{e}{p}_{n}\left(x\right){v}_{p}+2{q}_{e}{n}_{i}\left(x\right){v}_{i}$$

where ${q}_{e}$—elementary charge, $x$—positional coordinate,$t$– time $, {n}_{p}\left(x\right)$—the electron concentration in the p-region, ${p}_{n}\left(x\right)$– the hole concentration in the n-region, ${n}_{i}\left(x\right)$—the intrinsic concentration of charge carriers in the i-$region, {v}_{n}$, ${v}_{p}$, ${v}_{i}$—diffusion velocities of electrons, holes n, p and i-regions. The velocity of charge carriers in the corresponding areas ${v}_{n}=\frac{{L}_{n}}{{\tau }_{n}}$, ${v}_{p}=\frac{{L}_{p}}{{\tau }_{p}}$, ${v}_{i}=\frac{{L}_{i}}{{\tau }_{i}}$, where $L_{n} , L_{p} , L_{i}$—diffusion length of charge carriers in the corresponding region, the diffusion length for the corresponding region is defined as $L=$ $\sqrt{D\tau }$*, *${\tau }_{n}$*, *${\tau }_{p}$*,* ${\tau }_{i}$—lifetime of charge carriers in the corresponding region. The distribution of the concentration of charge carriers in the i-region obeys an exponential law, and can be expressed by the following expression (10)^28,29^.

3 $${n}_{i}\left(x\right)={n}_{i0}\left[1-exp\left(-\frac{{W}_{i}}{{L}_{i}}\right)\right]$$

where ${\mathrm{n}}_{\mathrm{i}0}$—the concentration of charge carriers in the i—region after drift, ${\mathrm{W}}_{\mathrm{i}}$—thickness of the i—region. Let’s apply Shockley's formula for Eq. (2). To do this, we divide the Shockley equation by the cross-sectional area of the detector and apply the definition of current density (4).

4 $$j\left(x,t\right)= {q}_{e}\left[{n}_{p}\left(x\right){v}_{n}+ {p}_{n}\left(x\right){v}_{p}+2{n}_{i}\left(x\right){v}_{i}\right]\left[exp\left(-\frac{qV}{kT}\right)\right]$$

where $k$—Boltzmann constant, $T$—absolute temperature. This current density will be established before the detection of radioactive decay particles. At the moment of detection, there is a sharp jump in current for a short period of time. Since the sensitive area of the detector is the i–region, electron–hole pairs are generated at the time of detection. We write down the Poisson continuity equation for electrons and holes at the time of detection, neglecting recombination (5, 6).

5 $$\frac{\partial {n}_{i}\left(x,t\right)}{\partial t}+{q}_{e}v\frac{\partial n\left(x,t\right)}{\partial x}=G(x,t)$$

6 $$\frac{\partial {p}_{i}\left(x,t\right)}{\partial t}+{q}_{e}v\frac{\partial n\left(x,t\right)}{\partial x}=G(x,t)$$

where $v = \mu {E}_{f}$ drift velocity of charge carriers, $\mu$—mobility of the corresponding charges, ${E}_{f}$—electric field. The solution of these partial differential equations will be the following general solutions:

7 $$\left\{\begin{array}{c}{n}_{i}=\int G\left(x,t\right)dt+{C}_{1}\\ {n}_{i}= \frac{\int G\left(x,t\right)dt+{C}_{2}}{{q}_{e}v}\end{array}\right.$$

where ${C}_{1}, {C}_{2}$– integration constants. The generation function can be expressed in terms of the energy of a radioactive particle and the energy required to generate one electron–hole pair as follows (8):

8 $$G\left(x,t\right)=\left[\frac{E}{\varepsilon {V}_{0}}-R(x)\right]\delta (t)$$

where *E* – the energy of a radioactive particle, *ε* – the energy required to create one electron–hole pair, $\varepsilon =\frac{14}{5}{E}_{g}+{\varphi E}_{R}$ ,^34,35^.* E*_*g*_—band gap of semiconductor material, *φE*_*R*_—optical phonon losses, *V*_*0*_ = *Sx*—unit of volume in which electron–hole pairs were generated, $\delta (t)$—Dirac delta function, *R*(*x*)—recombination function. Generation function has concentration units. Lost charge for recombination can be expressed as follows.

9 $${q}_{loss}= {q}_{0}\left(1-\lambda \right)$$

where *q*_*0*_—charge of generated electrons when a nuclear particle falls*,* $\lambda = \frac{{t}_{tr}}{\tau }$^37^—relative charge loss, *t*_*tr*_—time of plasma track, τ—lifetime of charge carriers. During time of plasma track high density of charged particles shields an external electric field. When the plasma track time interval ends all charge carriers are dispersed in the i-region. From charge value can be derived number of charged particles and their concentration, which depends on energy of nuclear particle and material of detector. Recombination function *R*(*x*), which also has units of concentration, can be expressed taking into account Eq. (9) as follows (10):

10 $$R\left(x\right)= \frac{\frac{E}{\varepsilon }{V}_{0}\left(1-\frac{{t}_{tr}}{\tau }\right)}{S\left(x\right)x}$$

where *V*_*0*_ – is initial volume where at the moment of generation all electron hole pairs located before recombination, ${V}_{0}=\pi {{r}_{tr}}^{2}{l}_{tr}$^36^, ${r}_{tr}$—track radius, ${l}_{tr}$—length of track of α-particle in detector, *S*(*x*)—function of flared base cylinder, *x*—coordinate.

We consider the initial conditions to be $x=d$, where d—coordinates of the i—region where electron hole pair generated, and t = 0. Then the initial concentration at the time of particle detection:

11 $${n}_{i}\left(d,0\right)= \frac{E}{\varepsilon {V}_{0}}$$

From here, integrating (8) and substituting the initial conditions (11) into the system (7), we obtain:

12 $${C}_{1}=\frac{E}{\varepsilon }\left(\frac{1}{{V}_{0}}-\frac{1-{V}_{0}(1-\lambda )}{Sx}\right), {C}_{2}=\frac{E}{\varepsilon }\left(\frac{{q}_{e}v}{{V}_{0}}-\frac{\mathit{ln}\left|x\right|}{S}\left(1-{V}_{0}\left(1-\lambda \right)\right)\right)$$

And finally, for the concentration of charge carriers in the i-region, we obtain:

13 $${n}_{i}=\frac{E}{\varepsilon }\left[\left(\frac{1}{S}-\frac{{V}_{0}}{S}(1-\lambda )\right)\left(\frac{1}{x}h\left(t\right)-\frac{1}{x}+\frac{1}{{q}_{e}v}\delta \left(t\right)ln\left|x\right|\right)+\left(\frac{1}{{V}_{0}}-\frac{ln\left|x\right|}{{q}_{e}vS}\left(1-{V}_{0}\left(1-\lambda \right)\right)\right)\right]$$

Then the total current through the detector consists of the current flowing through it before detection and the current through the detector at the time of detection (14).

14 $$I= {q}_{e}S\left[{n}_{p}\left(x,t\right){v}_{n}+ {p}_{n}\left(x,t\right){v}_{p}+2{n}_{i0}\left(x,t\right){v}_{i}\left[1-exp\left(-\frac{{W}_{i}}{{L}_{i}}\right)\right]\right]\left[exp\left(-\frac{{q}_{e}V}{kT}\right)\right]+{q}_{e}S{n}_{i}$$

**Table:** 

${n}_{p}$ = ${p}_{n}$ = ${n}_{i0}$	10^6^ particles/m^3^	${\tau }_{n}$ = ${\tau }_{p}$ = ${\tau }_{i}$	5·10^–4^ s
${D}_{n}$	31∙10^–4^ m^2^/s	${W}_{i}$	2∙10^–3^ m
${D}_{p}$	12∙10^–4^ m^2^/s	*T*	298.15 K
$S=\pi {r}^{2}$*, r* = *55 mm*	0.0095 m^2^	*U*	300 V
${\mu }_{n}$	0.15 m^2^/(V s)	${\mu }_{p}$	0.04 m^2^/(V s)

**Figure 2:**
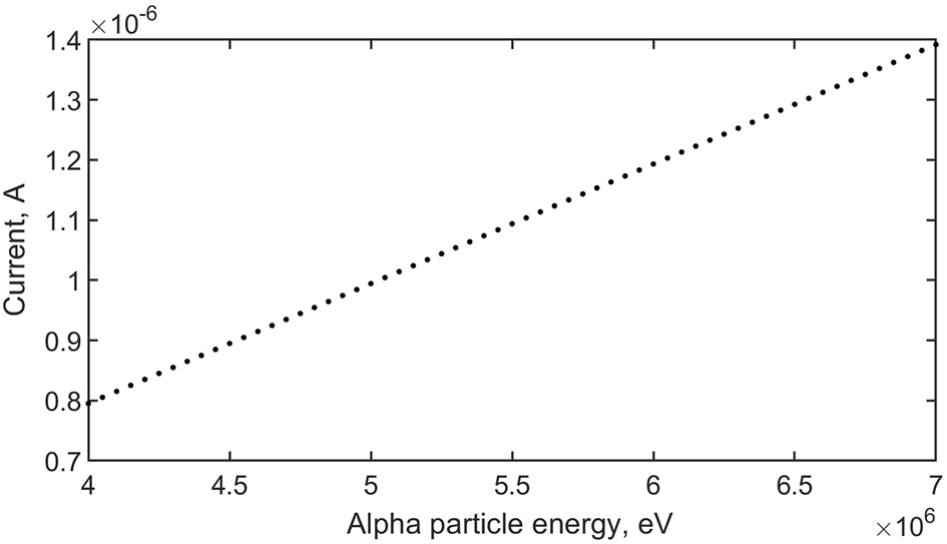
Dependence of current on radioactive particle energy.

**Figure 3:**
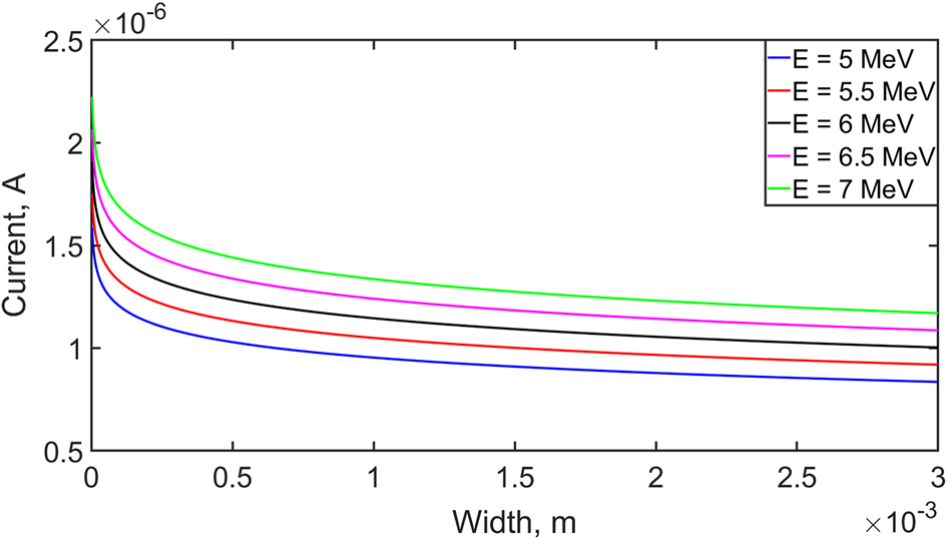
Dependence of current density on radioactive particle energy and detector thickness.

Figure 2 shows the dependence of the current flowing through a semiconductor detector at different energies of α-particles. Figure 3 shows the dependence of the current flowing through a semiconductor detector at different energies of α-particles on detector thickness.

Detector resolution FWHM (full width of the distribution at one half of its maximum height) is determined by Fano factor which is the ratio of the standard deviation of the created electron–hole pairs to the average value of electron–hole pairs (15)^38^.

15 $$F= \frac{\stackrel{-}{{\left(N-\overline{N }\right)}^{2}}}{\overline{N} }$$

where F—Fano factor, N—generated electron–hole pairs number, $\overline{\mathrm{N} }$—average generated electron–hole pairs number. Theoretical calculations of the Fano factor are given in the works^39–41^. For silicon factor Fano can be calculated using the following Eq. (16)^40^ :

16 $$F= {\left(\frac{\varphi {E}_{R}}{\varepsilon }\right)}^{2}+0.14\left(\frac{\frac{3}{2}{E}_{g}}{\varepsilon }\right)$$

In the works^42,43^ Fano factor is equal 0,117–0,118. In the work^44^ it is shown that Fano factor is decreasing by 5 times from 0.5 to 0.1 during last 40 years.

FWHM is determined from the Eq. (17):^38^

17 $$FWHM=2.36\sqrt{\varepsilon FE}$$

## Equivalent circuit of p–i–n detector

**Figure 4:**
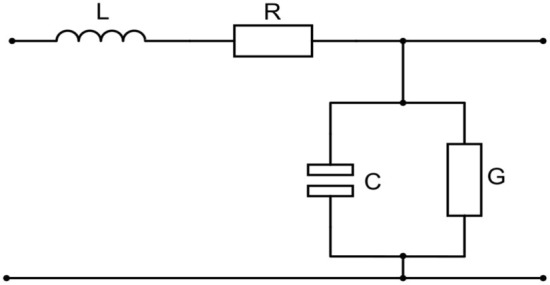
Equivalent circuit of the p–i–n detector.

To simulate p–i–n detectors of nuclear radiation under various conditions, an equivalent electrical circuit was obtained, which is based on the simulation results obtained above using the Shockley model. Let's imagine the p–i–n detector as a two-port network, as shown in Fig. 4.

Let's make a system of telegraph equations for this circuit (18).

18 $$\left\{ {\begin{array}{*{20}c} {u = \frac{1}{GR}\frac{{\partial^{2} u}}{{\partial x^{2} }} - \frac{LC}{{GR}}\frac{{\partial^{2} u}}{{\partial t^{2} }} - \frac{RC + GL}{{GR}}\frac{\partial u}{{\partial t}}} \\ {i = \frac{1}{GR}\frac{{\partial^{2} i}}{{\partial x^{2} }} - \frac{LC}{{GR}}\frac{{\partial^{2} i}}{{\partial t^{2} }} - \frac{RC + GL}{{GR}}\frac{\partial i}{{\partial t}}} \\ \end{array} } \right.$$

where u—the voltage on the detector at the moment of the fall of the charged particle, i—the current at the moment of the fall of the charged particle, R—the resistance of the detector, L—the equivalent inductance, C—the capacitance of the detector, G—the conductivity. Since in Eq. (12) we obtained the current, then in the future from system (18) we will use the second equation. We bring the second equation from the system (18) to the form (19).

19 $$i=aX+bY+cZ+d$$

where $\mathrm{a}=\frac{1}{GR}$*, *$b=\frac{LC}{GR}$*, *$c=\frac{RC+GL}{GR}$*, *$X=\frac{{\partial }^{2}i}{\partial {x}^{2}}$*, *$Y=\frac{{\partial }^{2}i}{\partial {t}^{2}}$*, *$Z=\frac{\partial i}{\partial t}$*, d*—an arbitrary constant.

**Figure 5:**
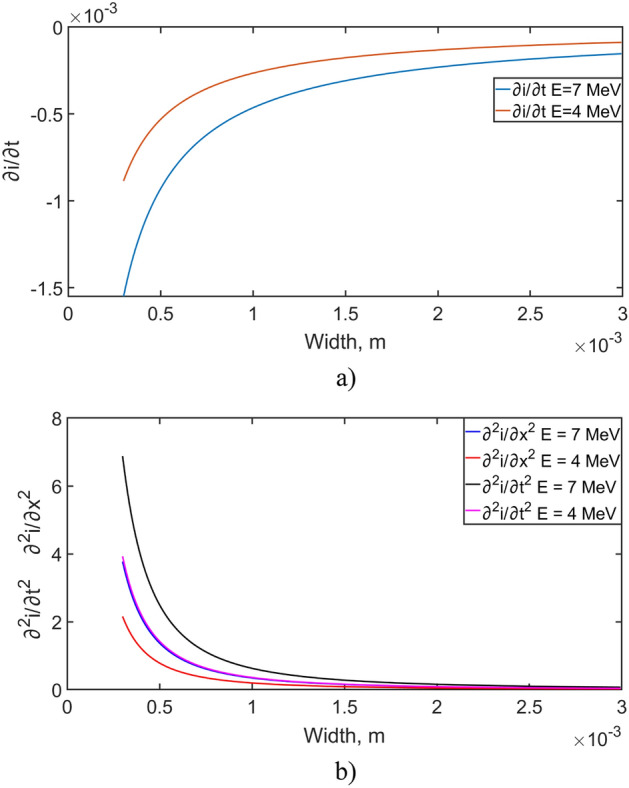
(**a**) First order partial derivatives of current in time, (**b**) second order partial derivatives of current in time and coordinate.

The left part of Eq. (14) is described by Eq. (12) and can be calculated for various parameters of this equation. In the right part there are partial derivatives of the second order of the current in the positional coordinate and in time, as well as a partial derivative of the first order in time. Consequently, having the values of the current depending on time and on the positional coordinate, partial derivatives of the first and second order can be numerically obtained with respect to the corresponding variables. Figure 5 shows the partial derivatives of the first (5a) and second order (5b) in time, as well as the second order in the positional coordinate for the α-particle energy of 4 MeV and 7 MeV.

The task of determining arbitrary variables a, b, c and d can be determined using the multiple regression method. At the same time, the coefficient of determination R^2^ of multiple regression is 0.98124. Substituting the coefficients obtained from the model, we obtain the expression (20).

20 $$i=\left(-99.02\pm 4.29\right)X+(54.33\pm 2.36) Y-(0.00137 \pm 8.43\cdot {10}^{-6}) Z+(1.71\cdot {10}^{-6}\pm 2.27\cdot {10}^{-7})$$

To return to the replaced variables and find the parameters of the equivalent circuit, we solve the following system (21), which follows from the replacement of variables for (19).

21 $$\left\{ {\begin{array}{*{20}c} {\frac{1}{GR} = - 99.02 \pm 4.29} \\ {\frac{LC}{{GR}} = 54.33 \pm 2.36} \\ {\frac{RC + GL}{{GR}} = - 0.00137 \pm 8.43 \cdot 10^{ - 6} } \\ \end{array} } \right.$$

**Figure 6:**
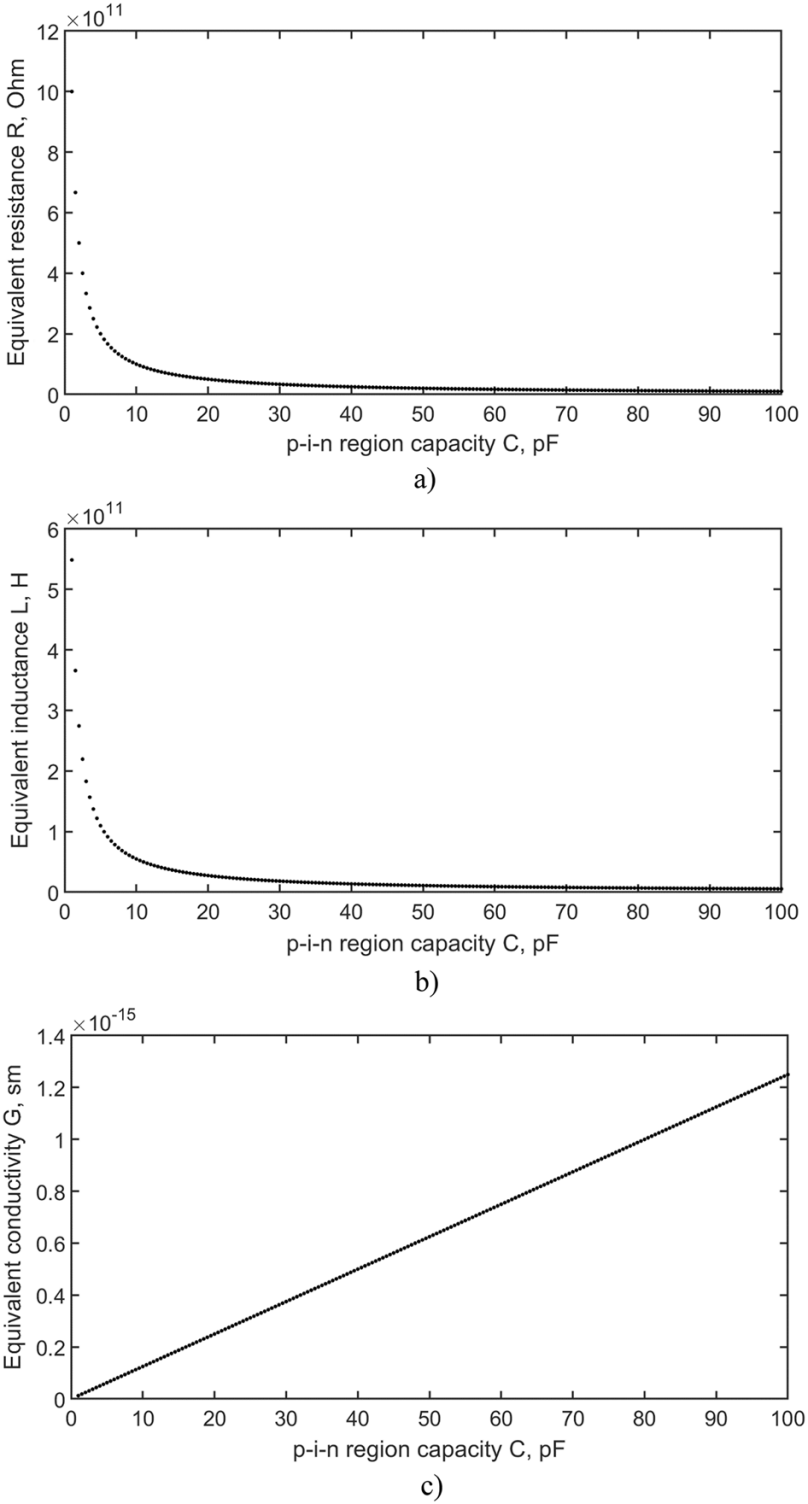
Parameters of the equivalent p–i–n detector circuit.

The system consists of three equations, but contains four variables. The capacitance of the p–i–n structure as shown in^28^ has an order of tens of pF, and depends on the inversely biased voltage. Then the values of R, L, G will depend on the capacity. Solving system of equations we obtain R, L, G for different capacitances of detector. Figure 6 shows the dependencies of the parameters R, L, G respectively of the equivalent circuit on the capacitance of the p–i–n diode.

The resulting equivalent circuit of a semiconductor p–i–n detector of nuclear radiation is useful in modeling the response of the detector to external influences in the construction of readout electronics.

**Figure 7:**
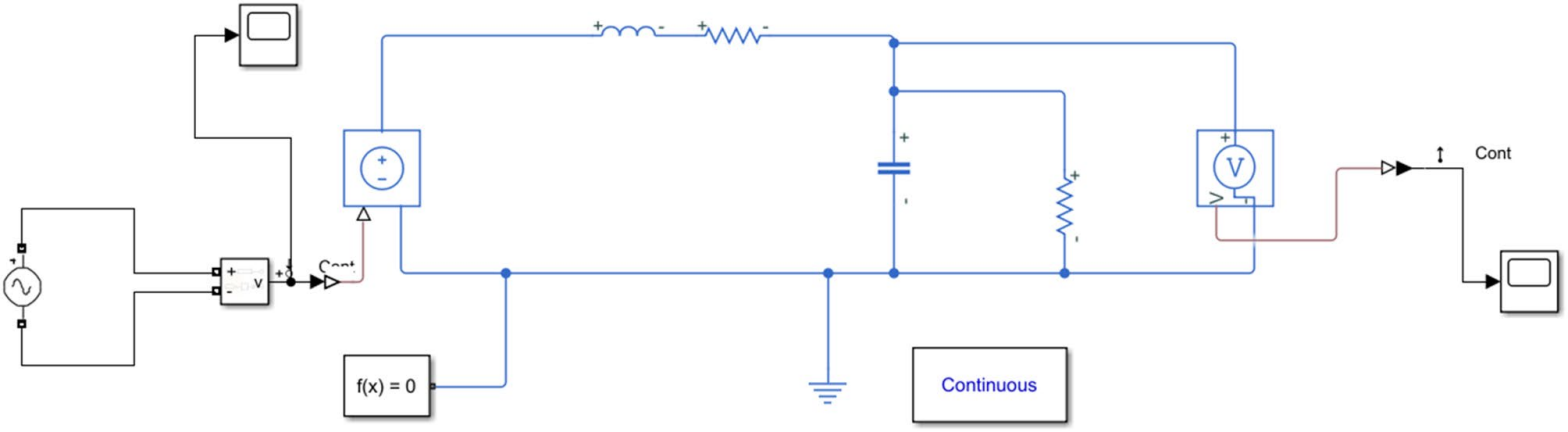
Modeling frequency response using MATLAB Simulink.

**Figure 8:**
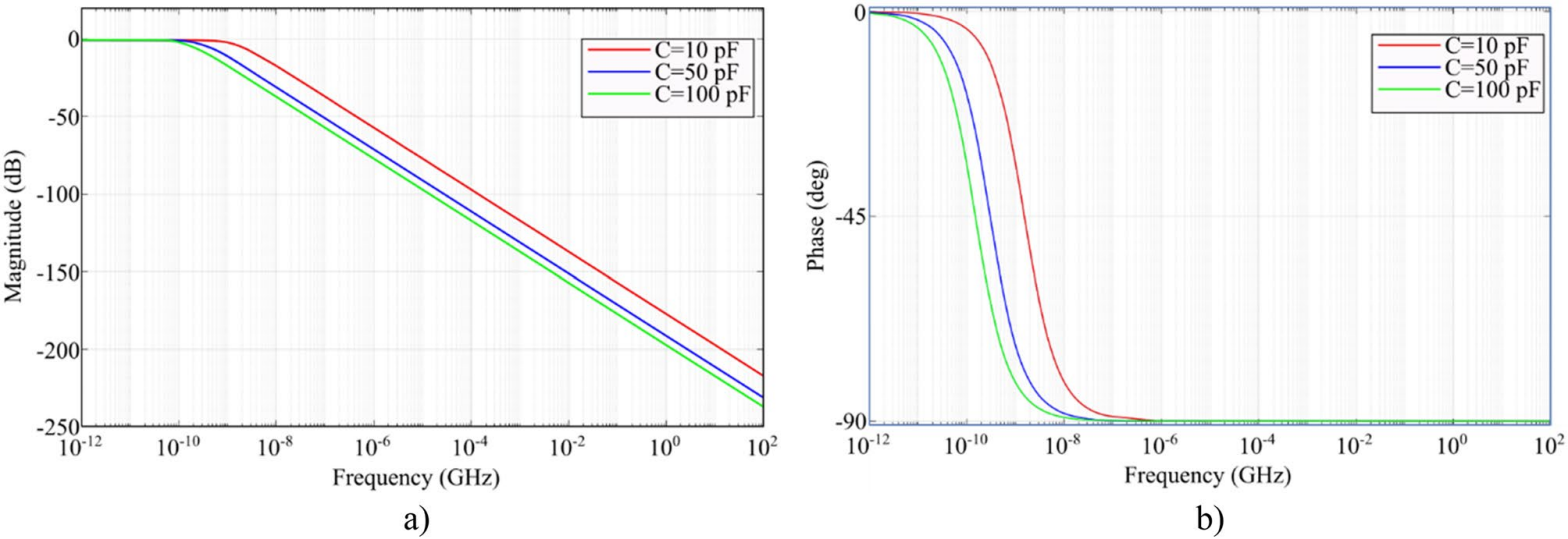
Frequency response of equivalent circuit.

Figure 7 shows an equivalent detector circuit made in MATLAB Simulink. Figure 8 shows the results of modeling the frequency response of an equivalent circuit. As can be seen from the graph, in the high frequency range up to several tens of gigahertz, the reaction is a linear function. At the same time, a phase shift of -90° is observed in this range.

## Modeling p–i–n detector using Monte Carlo method

**Figure 9:**
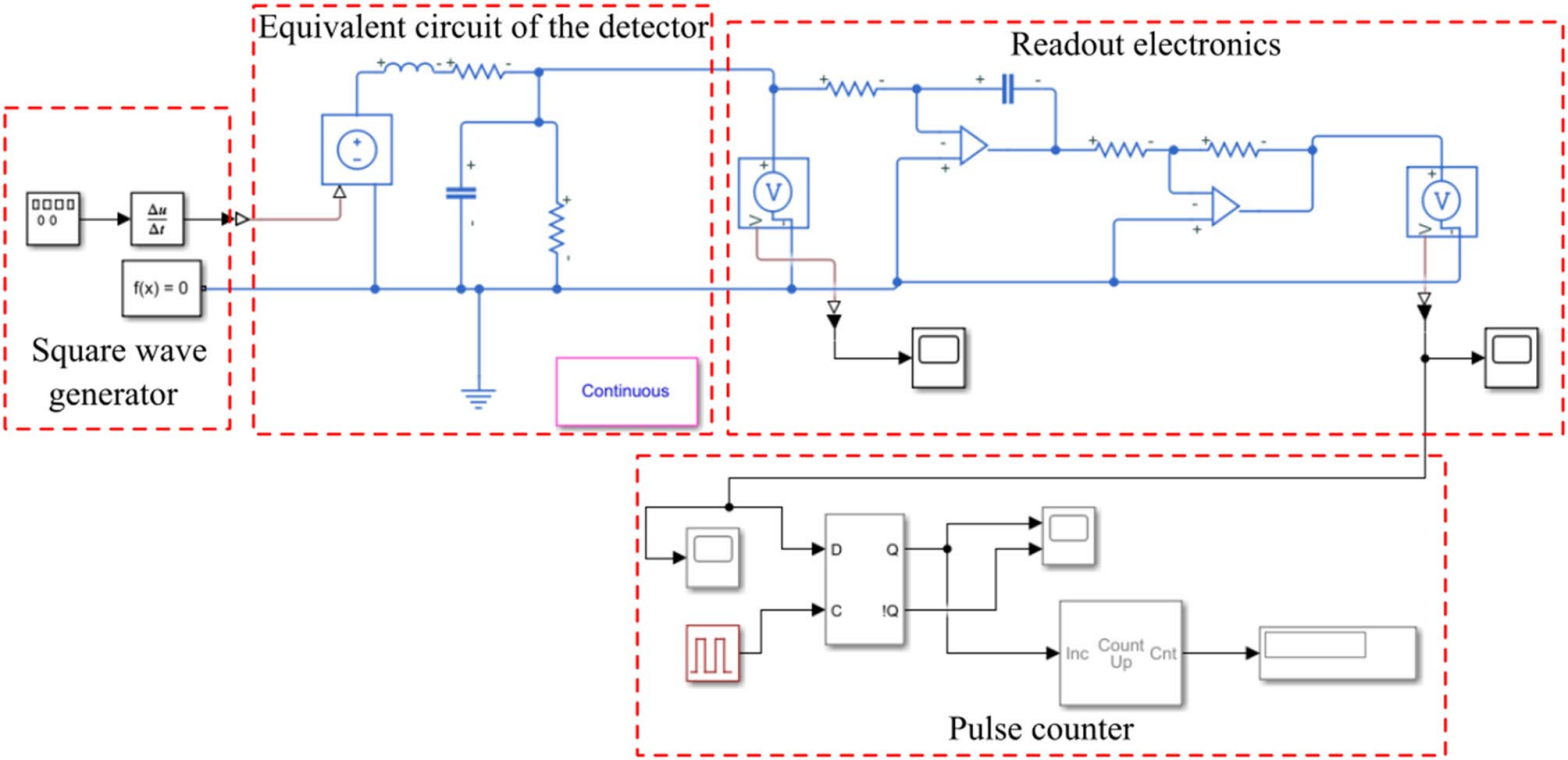
Equivalent circuit of the detector and readout electronics.

In the resulting equivalent circuit, it is necessary to add readout electronics to simulate the response of the detector at the time of particle detection. Figure 9 shows the equivalent circuit of the detector and readout electronics, made in MATLAB Simulink. The source of the signals is a block of rectangular pulses, followed by a block of a first-order derivative to create a Dirac function. Next, the signal passes through the equivalent detector circuit and the readout electronic unit, which is an integrating circuit and an amplifier. An integrating circuit is necessary to obtain the charge function as an integral of current and voltage as a function of charge. Thus, the voltage amplitude is proportional to the energy of the particles, and the number of particles is equal to the number of pulses detected by the circuit. The number of particles is determined using the pulse counter block.

To simulate the detector operation, we use the particle energy distribution during the alpha-decay of the uranium isotope ${}_{92}{}^{233}\mathrm{U}$, thorium isotope ${}_{90}{}^{227}\mathrm{Th}$ and americium isotope ${}_{95}{}^{241}\mathrm{Am}$. To apply the Monte Carlo method, we present the energy distribution function as a step function, adding up the probabilities of each event sequentially. We will generate random numbers from 0 to 1 and determine which probability interval this random number belongs to. Having determined the probability interval, we determine the corresponding energy level and, accordingly, the amplitude of the signal generator in the equivalent circuit.

**Figure 10:**
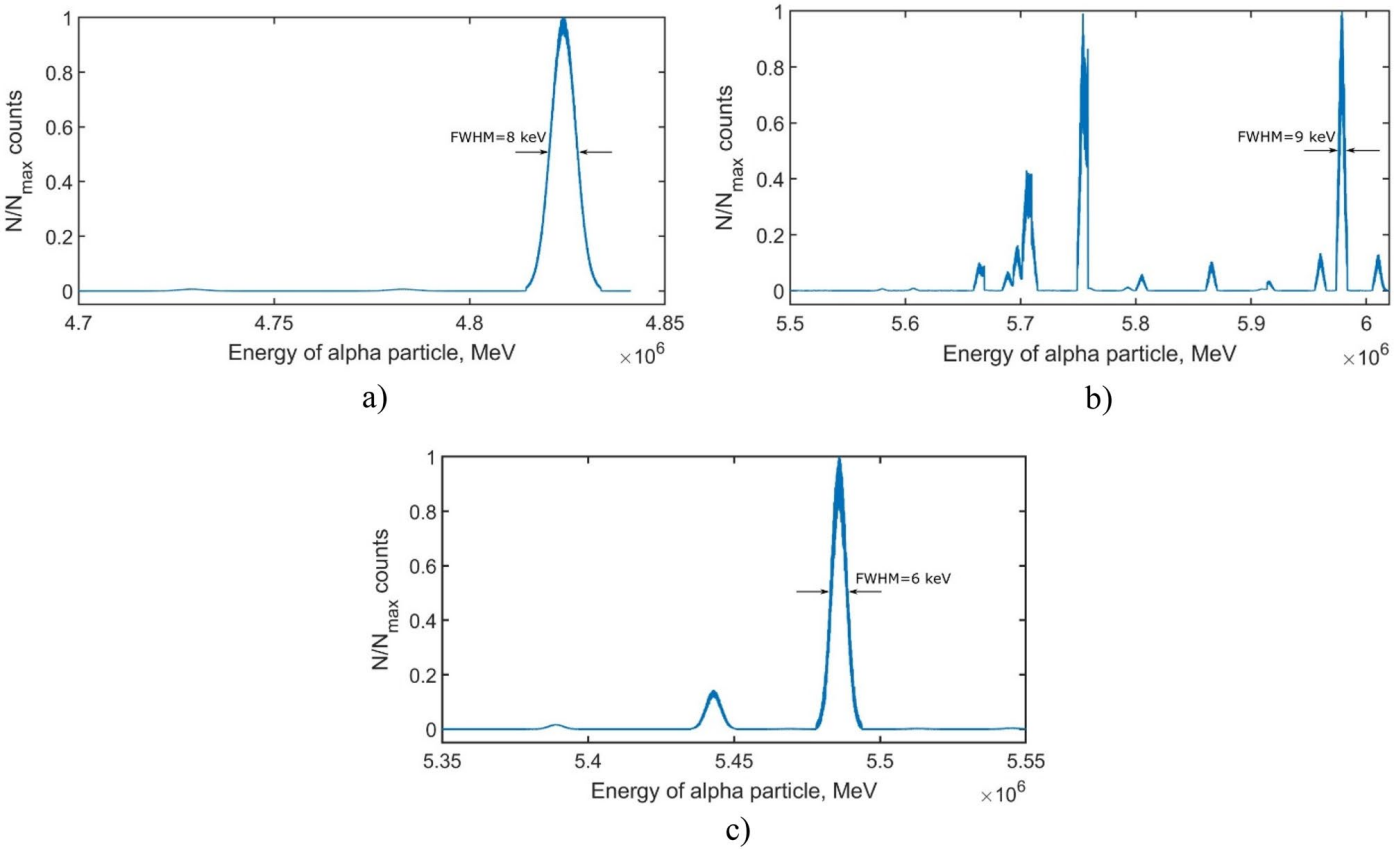
Alpha-decay spectra of (**a**) uranium isotope ${}_{92}{}^{233}\mathrm{U}$ (**b**) thorium isotope ${}_{90}{}^{227}\mathrm{Th}$ and (**c**) americium isotope ${}_{95}{}^{241}\mathrm{Am}$.

Performing this operation about 10^7^ times and counting the number of random numbers that fell on a particular probability interval, we will plot the dependence of the number of particles on the energy of the particles. Figure 10 shows alpha-decay spectra of a) uranium isotope ${}_{92}{}^{233}\mathrm{U}$ b) thorium isotope ${}_{90}{}^{227}\mathrm{Th}$ and c) americium isotope ${}_{95}{}^{241}\mathrm{Am}$. Alpha decay spectrum of uranium isotope ${}_{92}{}^{233}\mathrm{U}$ has one peak at energy 4.824 MeV with FWHM 8 keV, other peaks are at 4.783 MeV and 4.729 MeV. As can be seen from the alpha-decay spectrum of thorium, particles with an energy of about 5.98 MeV have the greatest contribution with FWHM = 9 keV, another peak is observed in the energy region of 5.76 MeV. Alpha decay spectrum of americium ${}_{95}{}^{241}\mathrm{Am}$ has three peaks at energies 5.486 MeV, 5.443 MeV and 5.389 MeV with FWHM = 6 keV.

## Discussion of the results

In the works^28,29^ generation of current totally determined by telegraph equation. Presented analytical solution obtained differential equations gives opportunity to judge about accumulated charge, however, the processes of recombination and ionization and the physical properties of the semiconductor structure are not taken into account, which is one of the most important problems in our work. It was reflected in Eqs. (1)–(4) and (8)–(10). The results of the simulation of the detector in the work^30^, in particular, the current strength of the order of microamperes is consistent with the results obtained in our model in Fig. 3. In this case, the value of the linear current depends on the energy of the charged particle, as shown in Fig. 2.

The simulation results of the obtained model using the Monte Carlo method show satisfactory results. The obtained alpha-decay spectra coincide with the experimental data shown in^45–47^. Theoretical calculations of FWHM closely related with calculation of Fano factor. Equation (16) obtained in the work^40^. In the work^48^ Fano factor obtained for silicon is equal 0.07. In our model Fano factor, calculated using Eq. (16) is equal 0.0895.

In our previous works^5,15^ the capacity of the developed detectors is of the order of tens of pF, the resistance of the detectors is of the order of tens of kΩ. Detector diameter 110 mm, thickness 8–10 mm. In this paper, the conductivity values G were calculated taking into account the approximation using multiple regression, as shown in Fig. 6, and have values of the order of 10^–15^ Sm, then the resistance of the i-region is of the order of 10^15^ Ω. The series resistance R and inductance L are also of the high order of 10^11^. This may be due to the use of the telegraph equation to describe the flow of electric current. The rapid signal decay that is observed when using the Shockley equation results in a rapid voltage drop. The rapid decrease in current in turn leads to high resistance, which is what we are seeing.

## Conclusion

As a result of the work, an equivalent circuit of a p–i–n nuclear radiation detector was obtained. The model is based on the Shockley diode equation for a semiconductor, without taking into account the effects associated with the interaction of the crystal lattice of the p–i–n structure, the effects of lithium ions in the i-region on the generation and movement of charge carriers. This approach makes it possible to simplify the model for linearization and the possibility of using the multiple regression method to obtain an equivalent detector circuit by identifying general trends in changes in physical quantities and without detailing the effects occurring in the nodes of the semiconductor crystal lattice. Using experimental data on particle energy distribution during the alpha-decay of the uranium isotope ${}_{92}{}^{233}\mathrm{U}$, thorium isotope ${}_{90}{}^{227}\mathrm{Th}$ and americium isotope ${}_{95}{}^{241}\mathrm{Am}$ the alpha-decay spectrum was obtained using the Monte Carlo method in relation to the proposed equivalent circuit. Obtained alpha-decay spectra coincides with the experimental data, presented in previous works of other authors.

## Data Availability

All data generated or analyzed during this study are included in this published article.
